# Impact of *MMP1*, *MMP2*, and *MMP3* Gene Variations on Susceptibility to Peyronie’s Disease and Clinical Progression

**DOI:** 10.3390/ijms27167194

**Published:** 2026-08-12

**Authors:** Gokhan Cevik, Arzu Ay, Nevra Alkanli, Hakan Akdere, Burak Akgul

**Affiliations:** 1 Department of Urology, Faculty of Medicine, Trakya University, Edirne 22030, Turkey; hakanakdere@trakya.edu.tr (H.A.); burakakgul@trakya.edu.tr (B.A.); 2 Department of Biophysics, Faculty of Medicine, Trakya University, Edirne 22030, Turkey; arzuay@trakya.edu.tr; 3 Department of Biophysics, Faculty of Medicine, Haliç University, Istanbul 34060, Turkey; nevraalkanli@halic.edu.tr

**Keywords:** Peyronie’s disease, matrix metalloproteinase, gene variation, PCR-RFLP, prognosis

## Abstract

The aim of this study was to determine the association between matrix metalloproteinase (*MMP1*, *MMP2*, and *MMP3*) gene variations and susceptibility to Peyronie’s disease, and to investigate their effects on disease progression. This cross-sectional case–control study included a total of 182 individuals—91 patients with Peyronie’s disease and 91 healthy controls. Peripheral venous blood samples (5 mL) were collected, and genomic DNA was isolated using standard spin-column extraction kits. The promoter regions of *MMP1*, *MMP2*, and *MMP3* were amplified via polymerase chain reaction (PCR), and genotyping was performed using restriction fragment length polymorphism (RFLP) assays with specific restriction endonucleases. All genotyping procedures were conducted in a blinded fashion. Hardy–Weinberg equilibrium analysis (HWE), binary logistic regression with rigorous multiplicity adjustments (Bonferroni-adjusted *p*-values), and non-parametric Kruskal–Wallis/Mann–Whitney tests with Dunn–Bonferroni post hoc comparisons were utilized. The frequency of the 2G allele for *MMP1* (−1607 1G/2G) was significantly higher in the patient group (0.4830) compared to the control group (0.2360; *p* < 0.05). Similarly, significant differences in allele frequencies were observed for *MMP2* (−735 C/T) and *MMP3* (−1171 5A/6A) between the cohorts (*p* < 0.05). HWE analysis confirmed that all investigated variations were in equilibrium in both groups (all *p* > 0.05). Logistic regression analysis showed that the *MMP1* 1G/1G genotype was associated with lower odds of Peyronie’s disease (OR: 0.250, *p* < 0.001), while the 1G/2G (OR: 2.032, *p* = 0.023) and 2G/2G (OR: 3.509, *p* = 0.005) genotypes were significantly associated with increased odds. Temporally, carriers of the *MMP1* 2G/2G genotype presented predominantly in the acute phase, exhibiting a significantly shorter median symptom duration (6.00 months, IQR: 3.00–9.00) compared to 1G/1G carriers (12.00 months, IQR: 5.25–36.00; *p* = 0.016, adj *p* = 0.048). For *MMP2* (−735 C/T), the CC genotype was associated with higher odds of Peyronie’s disease (OR: 3.049, *p* < 0.001), while the TT genotype was associated with lower odds (OR: 0.278, *p* = 0.002). Furthermore, penile plaques were detected in 90% of patients with the CC genotype (*p* < 0.001). Patients with the CC genotype presented with a median symptom duration of 6.00 months (IQR: 3.00–12.00), whereas TT carriers exhibited a significantly longer duration (24.00 months, IQR: 4.00–48.00; *p* = 0.002, adj *p* = 0.006). Regarding *MMP3* (−1171 5A/6A), the 5A/5A (OR: 3.930, *p* = 0.002) and 5A/6A (OR: 2.050, *p* = 0.022) genotypes were significantly associated with increased odds of the disease, while the 6A/6A genotype showed an inverse association (OR: 0.226, *p* < 0.001). Patients carrying the 5A allele presented characteristically in the acute phase, with a median symptom duration of 6.00 months (5A/5A) or 4.00 months (5A/6A), which was significantly extended to a median of 24.00 months (IQR: 7.50–54.00) in the 6A/6A genotype (*p* < 0.001, adj *p* < 0.001). The findings indicate robust statistical associations between the *MMP1* 2G, *MMP2* C, and *MMP3* 5A alleles and increased susceptibility to Peyronie’s disease, as well as distinct correlations with compressed symptom durations, acute-phase characteristics, and plaque presence. Because this study relied on peripheral blood DNA genotyping without direct tissue expression quantification in a cross-sectional design, these genetic variations reflect strong exploratory associations rather than direct causal evidence. These markers highlight significant prognostic potential that warrants validation in larger, prospective, longitudinal cohorts.

## 1. Introduction

Peyronie’s disease is a chronic connective tissue disorder characterized by the formation of fibrous plaques in the tunica albuginea of the penis, resulting in penile curvature, pain, and erectile dysfunction. The early stage is an active phase where affected individuals experience pain during erection and the onset of penile curvature. However, penile pain is not initially observed in all cases, with incidence rates varying between 20% and 70% among reported cases. The disease progresses to a painless chronic phase 12–18 months after onset, and a gradual worsening of penile curvature is observed in 20–50% of the cases. The changes occurring in the penis during disease progression rarely resolve spontaneously [1,2,3,4,5,6,7]. Furthermore, the prevalence of Peyronie’s disease can reach up to 20% depending on the population [8,9,10,11,12,13], and seriously impair the quality of life of patients, both physically and psychosocially [5,6,7]. The most widely accepted etiopathological theory is that repetitive microtraumas occurring during sexual activity in genetically predisposed individuals trigger a faulty and excessive wound healing process [14]. 

Nevertheless, current evidence indicates that susceptibility to Peyronie’s disease is polygenic and driven by fibrotic pathways, characterized by familial clustering and the involvement of multiple candidate genes or genetic variants. Although its precise genetic architecture continues to be elucidated, the fundamental pathological feature of Peyronie’s disease is the excessive accumulation of extracellular matrix (ECM) and the disruption of normal tissue structure. Matrix metalloproteinases (MMPs) play a critical role in ECM restructuring and homeostasis by breaking down structural proteins such as collagen and elastin. However, the balance between these enzymes and their natural inhibitors—the tissue metalloproteinase inhibitors (TIMPs)—is disrupted in the fibrotic plaques characteristic of Peyronie’s disease; MMP activity is suppressed by the action of pro-fibrotic cytokines, while the levels of TIMP-1 and PAI-1, which inhibit collagen degradation, are elevated. Genetic variations or aberrant expression of these enzymes can cause the collagen fibers to accumulate irregularly, leading to a loss of tissue elasticity and the formation of palpable, hard plaques. In addition, *MMP* gene variations can affect the transformation of fibroblasts into myofibroblasts and their migration within the tissue, leading to the expansion of the fibrotic focus [8,15,16,17,18,19,20,21].

The MMP family comprises a group of zinc-dependent endopeptidases that play a fundamental role in ECM remodeling, tissue repair, and various physiological processes. To date, at least 28 members of the MMP family have been identified in vertebrates, with 23 distinct genes expressed in humans. Based on their substrate specificity and structural domains, MMPs are broadly classified into collagenases, gelatinases, stromelysins, and membrane-type MMPs. Among this extensive family, MMP-1 (Interstitial Collagenase), MMP-2 (Gelatinase A), and MMP-3 (Stromelysin-1) represent critical regulators of the fibrotic cascade and are particularly relevant to the pathogenesis of Peyronie’s disease. Peyronie’s disease is characterized by abnormal fibrin deposition and excessive collagen accumulation (primarily Types I and III) within the tunica albuginea. MMP-1 serves as the primary initiator of fibrillar collagen degradation, cleaving the triple helix of collagen types I and III, which are highly overexpressed in Peyronie’s disease plaques. MMP-2 is responsible for further degrading gelatin (the cleaved product of collagen) and basement membrane components, playing a pivotal role in cellular migration and tissue remodeling. MMP-3 acts as a powerful activator of other latent MMPs (including pro-MMP-1) and degrades a wide range of ECM substrates such as proteoglycans, fibronectin, and laminin. Given that an imbalance in extracellular matrix degradation—traditionally regulated by the interplay between MMPs and their TIMPs—governs the progression from a localized inflammatory response to irreversible tunica albuginea fibrosis, identifying genetic markers that affect MMP activity is of paramount importance. Therefore, we anticipate that investigating the genetic variations within the *MMP1*, *MMP2*, and *MMP3* genes could provide critical insights into the genetic susceptibility and clinical progression of Peyronie’s disease [22,23,24,25].

The clinical course of Peyronie’s disease progresses from a painful acute phase to a stable chronic phase. Since MMPs are the primary enzymes responsible for the degradation of structural proteins like collagen and elastin, functional variations in their genes can critically affect the rate of ECM turnover. Specifically, single-nucleotide polymorphisms in the promoter regions of *MMP* genes (such as *MMP1*, *MMP2*, and *MMP3*) alter their transcriptional regulation, leading to either deficient or excessive enzymatic activity. In the context of Peyronie’s disease, such genetically driven imbalances in collagenolysis prevent proper tissue healing after microtrauma, accelerating the formation of dense, inelastic plaques. Consequently, these *MMP* genetic variations are considered significant molecular predisposing factors that may influence the progression, extent of fibrosis, and clinical severity of the disease [21,26,27]. Specific variations in *MMP* genes can directly affect the degree of penile curvature and plaque size by influencing the collagen turnover rate in the tissue. Furthermore, genetic differences in MMP expression following acute microtraumas determine the duration of inflammation and the extent of scarring. Deficient MMP activity can increase the calcification of plaques in the chronic phase, thereby warranting surgical intervention. Apart from genetic variations, systemic factors such as aging, diabetes, and smoking also modulate MMP expression and activity, as microRNAs can down-regulate (silence) or up-regulate (overstimulate) *MMP* genes either directly or via upstream regulators. In particular, the interaction between the TGF-beta signaling pathway and *MMP* genes is a key mechanism determining the progressive nature of Peyronie’s disease [20,28,29,30].

The aim of the present study was to investigate the distribution of *MMP* gene variations in individuals diagnosed with Peyronie’s disease, and their association with specific clinical parameters such as the disease stage and plaque localization. Within the framework of this detailed literature [7], the aim is to provide a basis for predicting the prognosis of Peyronie’s disease and developing targeted therapeutic strategies using MMP-focused genetic data.

## 2. Results

### 2.1. Descriptive Characteristics and Hardy–Weinberg Equilibrium (HWE) Analysis

The patient (n = 91) and control (n = 91) groups were standardized for age and major systemic comorbidities, and showed a homogeneous distribution of demographic variables. HWE analyses were performed to check the conformity of the genetic pool to population genetics principles, and the results confirmed that all three variations were in equilibrium. Pearson chi-square and probability values were calculated for both patient and control groups. For the *MMP1* (−1607 1G/2G) gene variation, Pearson chi2 and Pr were respectively 0.525 and 0.4687 in the patient group, and 2.878 and 0.0897 in the control group. For the *MMP2* (−735 C/T) gene variation, Pearson chi2 and Pr were respectively 2.201 and 0.1379 in the patient group, and 3.163 and 0.0753 in the control group. For the *MMP3* (−1171 5A/6A) gene variation, Pearson chi2 and Pr were respectively 0.89 and 0.3455 in the patient group, and 0.746 and 0.3877 in the control group. The Pr values for both the patient and control groups were above the 0.05 threshold at all loci, which confirmed lack of selection bias in the sample population and appropriate distribution of the alleles (Table 1).

### 2.2. MMP Gene Variations and Their Association with Disease Susceptibility

Univariate logistic regression models were established to compare the genotype distribution of *MMP1*, *MMP2*, and *MMP3* variations between the patient and control groups. As shown in Table 2, all three gene variations showed a significant association with the susceptibility to Peyronie’s disease (*p* < 0.001).

A significant proportion of healthy controls (61.5%, n = 56) had the homozygous wild-type 1G/1G genotype of the *MMP1*-1607 promoter region variation, and its frequency among the patients was only 28.6% (n = 26). Furthermore, the 1G/1G genotype was significantly associated with lower odds of Peyronie’s disease (OR: 0.250, 95% CI: 0.134–0.465, *p* < 0.001). The 1G/2G genotype was observed more frequently in patients compared to healthy controls (46.1% vs. 29.7%) and was significantly associated with a two-fold higher likelihood of the disease (OR: 2.032, 95% CI: 1.104–3.740, *p* = 0.023. The 2G/2G genotype of the *MMP1* gene was associated with a 3.5-fold increase in the odds of Peyronie’s disease (OR: 3.509, 95% CI: 1.476–8.343, *p* = 0.005; Table 2).

The C/T variation in the *MMP2* promoter region showed an asymmetric distribution between the two groups (*p* < 0.001). The frequency of the CC genotype, which is associated with high levels of the enzyme, was 54.9% (n = 50) in the patient group, compared to 28.6% (n = 26) in the control group. Furthermore, the presence of the CC genotype was significantly associated with a 3-fold higher odds of Peyronie’s disease (OR: 3.049, 95% CI: 1.649–5.635, *p* < 0.001). The CT genotype was detected at relatively similar frequencies in the patient (34.1%) and control (40.7%) groups, and did not show a statistically significant association with Peyronie’s disease (OR: 0.754, 95% CI: 0.413–1.377, *p* = 0.358). The TT genotype, which corresponds to low enzyme activity, was observed in 30.7% (n = 28) of the healthy controls, compared to only 11% (n = 10) of the patients. In the regression analysis, the TT genotype was significantly associated with lower odds of the disease (OR: 0.278, 95% CI: 0.126–0.614, *p* = 0.002) (Table 2).

The genotypic variations in the *MMP3* promoter region, which determines stromelysin-1 activity, showed the most significant differences (*p* < 0.001). The 5A/5A genotype was observed in only 8.8% (n = 8) of the controls, and its frequency increased to 27.5% (n = 25) in the patient group, which was significantly associated with approximately a 4-fold higher odds of Peyronie’s disease (OR: 3.930, 95% CI: 1.664–9.280, *p* = 0.002). The 5A/6A genotype was also more frequent in the patient population (45%) compared to the controls (28.6%) and was significantly associated with a 2-fold higher odds of Peyronie’s disease (OR: 2.050, 95% CI: 1.109–3.789, *p* = 0.022). The frequency of the 6A/6A genotype was 62.6% in the healthy controls compared to 27.5% in the patients, and the presence of this genotype significantly minimized the probability of disease (OR: 0.226, 95% CI: 0.121–0.423, *p* < 0.001) (Table 2).

The genotypic distribution of the *MMP* gene variations was also reflected at the allele level. The frequency of the 2G allele of the *MMP1* gene variation was 0.4830 (n = 88) in the patient group, compared to 0.2360 (n = 43) in the healthy controls. The frequency of the C allele, which is associated with the pathological *MMP2* gene variant, was 0.72 (n = 131) among the patients and 0.4890 (n = 89) among the healthy controls. The frequency of the *MMP3* 5A allele was 20.4% (n = 42) in the healthy control group, and increased significantly to 50% (n = 91) in the patients, highlighting a substantial genetic association with Peyronie’s disease (Table 1).

### 2.3. Correlation Between the Genotypic Distribution of MMP Gene Variations and the Presence of Fibrotic Plaques

The patients with Peyronie’s disease were divided into two phenotypic subgroups based on the presence (n = 78) and absence (n = 13) of plaques at clinical presentation. There was no statistically significant relationship between the plaque status at clinical examination and the genotype distribution of *MMP1* (*p* = 0.852, adj *p* = 1.000) and *MMP3* (*p* = 0.536, adj *p* = 1.000) gene variations. The percentage of patients with plaques was similar across the different genotypes for both the *MMP1* locus (88.5% for 1G/1G, 83.3% for 1G/2G, 87% for 2G/2G) and the *MMP3* locus (92% for 5A/5A, 85.4% for 5A/6A, 80% for 6A/6A). However, the *MMP2* (−735 C/T) variation correlated robustly and significantly with the presence of fibrotic plaques even after multiplicity correction (*p* < 0.001, adj *p* < 0.001). Mature fibrotic plaques were detected on clinical examination in 90% (n = 45) of the patients with the CC genotype, who have a high enzyme production phenotype, and in 93.5% (n = 29) of the patients with the CT genotype. In contrast, the majority of patients carrying the TT genotype—which is associated with low enzyme production—did not exhibit plaque formation at the time of clinical presentation (60%, n = 6; Table 3).

### 2.4. Genotype Distribution of MMP Variations According to Clinical Disease Stages

Based on the symptom duration, the disease was clinically classified into the acute/active inflammatory phase (≤12 months) and the chronic/stable fibrotic phase (>12 months). Although an unadjusted difference was observed in the distribution of *MMP1* genotypes between the two clinical stages (*p* = 0.026), this association approached significance after correction for multiple comparisons (adj *p* = 0.078). While 26.9% (n = 7) of the 1G/1G carriers presented to the clinic in the late/chronic phase, all patients (100%, n = 23) with the 2G/2G genotype were diagnosed while in the active/acute phase with severe symptoms. In addition, the genotype distributions for *MMP2* gene variation correlated robustly and significantly with the disease stage even after multiplicity adjustment (p = 0.001, adj *p* = 0.003). All patients with the CC genotype (100%, n = 50) presented with active/acute phase characteristics, while 40% (n = 4) of the patients with the TT genotype, which is associated with the stable chronic phase, presented with stable chronic phase characteristics. The most striking and robust association was detected at the *MMP3* locus (*p* < 0.001, adj *p* < 0.001). All 66 patients (100%) carrying the 5A allele—whether homozygous (5A/5A, n = 25) or heterozygous (5A/6A, n = 41)—presented to the clinic within the first 12 months of the disease (acute phase). In contrast, all patients at the chronic stable-stage (n = 9) had the 6A/6A genotype (36%) (Table 4). 

### 2.5. Genotype Distribution of MMP Variations According to Symptom Duration

To test the accuracy of categorical phase analyses using continuous variables and to identify precise duration trends free from outliers, a non-parametric Kruskal–Wallis test was applied to symptom durations on a monthly basis. Since not all data points showed a normal distribution, median and interquartile range (IQR) values are presented. The symptom duration showed significant differences depending on the *MMP1* genotype, which remained significant after correction for multiple comparisons (*p* = 0.016, adj *p* = 0.048). The median symptom duration to outpatient presentation was 12 months (IQR: 5.25–36) for individuals with the 1G/1G genotype for the *MMP1* gene variation, 8 months in patients with the 1G/2G genotype, and 6 months (IQR: 3–9, *p* = 0.016 vs. 1G/1G) in patients with the 2G/2G genotype. The genotype distribution of the *MMP2* gene variation also had a robust and significant impact on symptom duration (*p* = 0.002, adj *p* = 0.006). In patients with the CC genotype, the median time to clinical presentation was 6 months (IQR: 3–12), compared to 24 months in patients with the TT genotype (IQR: 4–48, *p* = 0.002, adj *p* = 0.006). In time-based non-parametric analyses, the sharpest and clearest chronological separation was confirmed for the *MMP3* gene variation (*p* < 0.001, adj *p* < 0.001). Patients with the 5A/5A (median: 6 months, IQR: 3.5–12) and 5A/6A (median: 4 months, IQR: 3–9.5) genotypes sought medical attention significantly earlier than the other groups (*p* < 0.001). In contrast, rigorous biostatistical post hoc comparisons (Dunn–Bonferroni test) revealed that patients carrying the 6A/6A genotype, which is associated with a stable disease course, had a significantly longer median symptom duration of 24 months (IQR: 7.5–54, *p* < 0.001, adj *p* < 0.001; Table 5).

## 3. Discussion

Peyronie’s disease is a localized fibrotic disorder developing within the tunica albuginea layer of the penis, characterized fundamentally by the uncontrolled and aberrant accumulation of ECM components. Although repetitive micro-traumas are widely implicated as the primary etiological trigger, the clinical observation that similar traumatic events do not induce Peyronie’s disease in every exposed individual points toward underlying genetic susceptibility factors [20,21]. In the present study, we evaluated the statistical associations between promoter and functional gene variations in *MMP1*, *MMP2*, and *MMP3* and the clinical presentation and temporal progression of Peyronie’s disease, integrating comprehensive phenotypic parameters while exercising rigorous methodological and multiplicity controls.

MMPs act as core enzymatic regulators of tissue remodeling, degradation, and wound healing. Interstitial collagenase MMP-1 is primarily responsible for initiating the breakdown of fibrillar type I and type III collagen fibers, thus setting the pace for early-stage tissue destruction and repair [31]. Our findings demonstrate a strong statistical association between the *MMP1* 2G allele and an aggressive, rapidly progressive disease phenotype. In the literature, the 2G insertion has been described as creating a putative binding site for the ETS transcription factor family, which is hypothesized to upregulate baseline transcriptional activity [31]. While our study did not measure direct tissue-level protein expression or enzymatic activity, we observed that individuals carrying variant genotypes presented predominantly within the active, highly inflammatory acute phase, exhibiting a significantly compressed median symptom duration of 6.00 months (IQR: 3.00–9.00) compared to 12.00 months (IQR: 5.25–36.00) for wild-type individuals (*p* = 0.016, adj *p* = 0.048). This statistical correlation suggests a clinical phenotype characterized by rapid progression and severe localized pain and deformity, aligning with previously proposed functional hypotheses regarding early matrix turnover.

Similarly, MMP-2 (gelatinase A) plays a critical role by degrading basement membrane constituents and denatured collagens, thereby facilitating fibroblast migration, survival, and phenotypic transformation into active myofibroblasts [20,28,29,30,32]. Mechanistically, overactivation of the TGF-β1 signaling pathway leads to aberrant MMP-2 expression and disrupts its stoichiometric balance with TIMP-2, resulting in disordered cross-linking of collagen fibrils [20,33,34]. The pronounced statistical predominance of the high-expression-associated *MMP2* CC genotype in our patient cohort, alongside its robust correlation with early plaque detection, suggests that this genetic variant is associated with the clinical stabilization of palpable fibrotic plaques within the tunica albuginea. In contrast, the TT genotype was statistically associated with lower odds of the disease (OR: 0.278), suggesting a potential inverse relationship rather than a definitive protective effect given the cross-sectional design. Although direct pathway activation (such as via TGF-β1 signaling or stoichiometric imbalances with TIMPs) was not quantified in our cohorts, the models in the literature suggest that such variations may influence local remodeling dynamics. Consistent with our statistical findings, patients with the CC genotype presented to the clinic with a median symptom duration of 6.00 months (IQR: 3.00–12.00), while those with the TT genotype remained symptomatic for 24.00 months (IQR: 4.00–48.00) (*p* = 0.002, adj *p* = 0.006). These associations imply that host *MMP2* genotype profiles correlate closely with the clinical velocity of the fibrotic process, pending direct tissue expression verification.

MMP-3 (stromelysin-1) broadens the proteolytic spectrum by degrading core matrix glycoproteins such as proteoglycans, fibronectin, and laminin, while additionally cleaving and activating other latent pro-enzymes like pro-MMP-9 [29,30]. The 5A allele located in the *MMP3* promoter region has been reported in prior literature to enhance transcription by disrupting binding sites for transcriptional repressors, resulting in potentially higher enzymatic output compared to the 6A allele [35]. Our clinical data align with this established context: patients carrying high-activity-associated 5A alleles presented almost universally during the early acute phase following symptom onset. Based on theoretical models, elevated MMP-3 levels could theoretically accelerate initial ECM degradation following trauma and synchronize acute inflammation with myofibroblast recruitment [20,29,30,35]. Statistically, the median symptom duration in patients with the 5A/5A (6.00 months, IQR: 3.50–12.00) or 5A/6A (4.00 months, IQR: 3.00–9.50) genotypes was very short, and this period was extended to 24.00 months (IQR: 7.50–54.00) in the 6A/6A genotype, which is associated with low transcriptional activity (*p* < 0.001, adj *p* < 0.001). While these mechanistic pathways remain putative inferences derived from literature precedent rather than direct proofs measured in our study, the statistical stratification across temporal phases is highly robust.

Taken together, these findings indicate that the *MMP1* 2G, *MMP2* C, and *MMP3* 5A alleles statistically construct a profibrotic genetic profile that correlates with accelerated disease manifestation and distinct clinical phenotypes in Peyronie’s disease. Similar enzymatic dysregulations are known from literature to compromise tissue compliance in other fibrotic disorders, such as cardiovascular remodeling [30]. Clinically, these observations highlight that genetic profiling of matrix-degrading pathways holds strong translational potential as a prognostic marker to differentiate rapidly progressive inflammatory phenotypes from slow-progressing, stable chronic forms.

Despite these insights, several limitations must be considered when interpreting our findings. First, while our sample size achieved adequate statistical power for single-locus evaluations, the cross-sectional design precludes definitive causal determinations, necessitating prospective longitudinal studies to establish genuine predictive sensitivity and specificity. Second, our analyses relied strictly on peripheral blood DNA genotyping without quantifying direct tissue-level mRNA transcription, protein expression, or enzymatic activity within the tunica albuginea; thus, mechanistic explanations remain hypothesis-generating based on the prior literature. Third, due to prompt care-seeking behaviors driven by painful acute symptoms, our cohort exhibited a heavy skew toward early-stage presentations, limiting our ability to track long-term chronic parameters such as plaque calcification. Finally, while appropriate multiplicity adjustments were rigorously applied to protect against Type I error inflation during subgroup comparisons, evaluating complex polygenic interactions via multilocus genetic profiling (MGP) will require much larger, multi-center cohorts to prevent statistical power dilution and unstable risk estimations. Overall, our study establishes robust genetic associations that provide a foundation for future mechanistic and longitudinal validation.

## 4. Materials and Methods

### 4.1. Selection of Patient and Control Groups

The current study was conducted in accordance with the principles of the Helsinki Declaration and approved by the Trakya University Faculty of Medicine Non-Interventional Clinical Research Ethics Committee (TÜTF-GOBAEK 2023/123, Decision No: 06/34, Date: 10 April 2023). Participants were informed in detail about the scope, purpose, procedures of the research, and their right to withdraw from the study at any stage without any sanctions. All participants were required to provide written informed consent. Based on a study conducted by Rodrigues et al., the total sample size was determined to be 170—at least 85 subjects in each group—with a 5% margin of error and an 80% power rating [36]. To improve computational reliability and tolerate potential loss of clinical data, we included 91 patients with Peyronie’s disease and 91 healthy controls (n = 182). To ensure the independence of the genetic/clinical data, the familial history of all participants was evaluated. All 91 patients included in the present study were genetically unrelated, and all cases were confirmed to be isolated with no evidence of family clustering. The patient group consisted of 91 consecutive male individuals who presented to the Department of Urology, Faculty of Medicine, Trakya University, and were diagnosed with Peyronie’s disease clinically and radiologically. A definitive diagnosis of Peyronie’s disease is made by a specialist urologist based on a comprehensive medical and sexual history, a detailed physical examination revealing the presence of palpable fibrotic plaques in the tunica albuginea, and objective documentation of penile deformity or curvature (≥15°) during erection. All patients underwent high-resolution penile color Doppler ultrasonography to assess plaque localization, size, and the presence of calcification. Patients diagnosed with stable or active stage Peyronie’s disease, characterized by palpable plaque and subjective/objective penile curvature, who were 19 years of age and older, volunteered to participate in the study, and provided written informed consent were included in the present study. Patients with congenital penile curvature, isolated erectile dysfunction without palpable fibrotic plaques, a history of penile fracture, or a history of major urological surgery were excluded from the study. Furthermore, in accordance with molecular epidemiological screening standards applied to eliminate systemic confounding factors that could alter MMP expression levels, individuals with chronic liver or kidney disease, active systemic infection, chronic inflammatory or autoimmune disorders, and those receiving systemic immunomodulatory or corticosteroid therapy were strictly excluded from the cohort. The healthy control group consisted of 91 male volunteers who presented to the urology or other outpatient clinics of the same hospital with non-inflammatory, non-fibrotic, or non-malignant conditions. Inclusion criteria for the healthy control group required participants to be ≥19 years old, appropriately matched with the patient cohort, and without personal or family history of Peyronie’s disease, Dupuytren’s contracture, or Ledderhose’s disease. Furthermore, physical examination confirmed the absence of palpable tunica albuginea plaques or clinical signs of penile deformity and curvature in the healthy controls. The exclusion criteria for the control group were the same as those applied to the patient group. Individuals with liver or kidney failure, chronic inflammatory phenotypes, active infections, or immune system disorders were systematically excluded to provide a homogeneous and unbiased biological background for the comparative analysis of *MMP* gene variations. To ensure data integrity and prevent information bias, all genotyping analyses were performed in a double-blinded manner, with laboratory personnel unaware of the patients’ clinical stages or symptom durations. All dataset entries were cross-checked independently by two investigators to verify accuracy and screen for potential data entry errors.

### 4.2. Selection of Genes and Variations

The candidate genes (*MMP1*, *MMP2*, and *MMP3*) and their respective variations were selected based on their established biological roles in ECM degradation and their documented involvement in Peyronie’s disease. The specific variations within the promoter regions were chosen due to their functional impact on transcription, such as the *MMP1* −1607 1G/2G (rs1799750) variation, which was selected because the insertion of an extra guanine (2G allele) creates an ETS transcription factor-binding site, significantly up-regulating promoter activity and increasing transcription. The *MMP2* −735 C/T (rs2285053) variation was chosen because the C-to-T transition disrupts a stimulating protein-1 (Sp1) binding site, leading to a substantial decrease in promoter activity and subsequent MMP-2 expression. The *MMP3* −1171 5A/6A (rs3025058) variation was included because the 5A allele exhibits a significantly higher transcription rate than the 6A allele due to differences in transcription factor-binding affinity, thereby directly modulating MMP-3 expression levels. Given these functional characteristics, these promoter variations serve as excellent candidates for evaluating genetic susceptibility [22,23,24,25,35].

### 4.3. Blood Sampling and DNA Extraction

Peripheral venous blood samples (5 mL) were collected from all patients and control subjects in EDTA-anticoagulated blood collection tubes (Vacutainer, Becton Dickinson, Franklin Lakes, NJ, USA). Samples were immediately processed or stored at −80 °C until genomic DNA extraction. Genomic DNA was isolated from peripheral blood leukocytes using a standard commercial spin-column DNA extraction kit (QIAamp DNA Blood Mini Kit, Qiagen, Hilden, Germany) in accordance with the manufacturer’s protocol. The concentration and purity of the isolated genomic DNA were determined by spectrophotometric optical density (OD) measurements, ensuring an OD260/280 ratio between 1.8 and 2.0. All molecular analyses were performed in the research laboratories of the Department of Biophysics, Trakya University and Haliç University. 

### 4.4. PCR-RFLP and Genotyping Quality Control

Genotyping of the functional single-nucleotide variations in the promoter regions of *MMP1* (−1607 1G/2G, rs1799750), *MMP2* (−735 C/T, rs2285053), and *MMP3* (−1171 5A/6A, rs3025058) genes was performed using the PCR-RFLP method (PCR; Techne TC–300, Techne, Cambridge, UK). Although high-throughput platforms like TaqMan^®^ SNP assays are widely available, PCR-RFLP was chosen as a highly reliable, cost-effective, and well-established alternative for laboratories analyzing a targeted, low number of specific loci.

To ensure the accuracy and reproducibility of the genotyping results, rigorous quality control procedures were implemented. First, laboratory personnel performing the genotyping were blinded to the clinical characteristics and group allocation of the patients. Second, negative controls (reaction mixtures containing molecular-grade water instead of template DNA) were included in each PCR run and restriction enzyme digestion batch to monitor for any potential contamination. Third, a randomly selected 10% of the DNA samples were subjected to repeated PCR-RFLP analysis by an independent operator, which demonstrated 100% concordance with the original genotyping results.

The PCR mix consisted of 50 ng DNA, 0.2 mM dNTPs, 1x PCR buffer, 3 mM MgCl_2_, 1.25 units of Taq DNA polymerase, and gene-specific forward (FP) and reverse (RP) primers (Invitrogen, Thermo Fisher Scientific, Waltham, MA, USA; Fermentas-Invitrogen, Thermo Fisher Scientific, Waltham, MA, USA). The primer sequences for *MMP1* (rs1799750) were FP: 5′-TCGTGAGAATGTCTTCCCATT-3′ and RP: 5′-TCTTGGATTGATTTGAGATAAGTCATATC-3′. The PCR cycle consisted of initial denaturation at 94 °C for 5 min, followed by 35 cycles of denaturation at 94 °C for 20 s, annealing at 55 °C for 20 s, and extension at 72 °C for 20 s; the reaction was completed with a final extension step at 72 °C for 5 min. The amplicon is 113 base pairs (bp) long. *MMP2* (rs2285053) was amplified using the primers FP: 5′-GGATTCTTGGCTTGGCGCAGGA-3′ and RP: 5′-GGGGGCTGGGTAAAATGAGGCTG-3′. Amplification conditions included an initial denaturation at 94 °C for 5 min, and denaturation at 95 °C for 45 s, annealing at 55 °C for 45 s, and extension at 72 °C for 45 s for 35 cycles; the reaction was completed with a final extension step at 72 °C for 10 min. The target PCR product is 391 bp in length. *MMP3* (rs3025058) was amplified using the primers FP: 5′-CTTCCTGGAATTCACATCACTGCCACCACT-3′ and RP: 5′-GGTTCTCCATTCCTTTGATGGGGGGAAAAA-3′. PCR parameters included an initial denaturation at 94 °C for 3 min, followed by denaturation at 94 °C for 50 s, annealing at 58 °C for 50 s, and extension at 72 °C for 20 s for 35 cycles; the reaction was terminated with a final extension step of 5 min at 72 °C. The target PCR product is 129 bp in length (Supplemental Files S1–S5). 

Following PCR validation, the amplified DNA fragments were digested overnight using specific restriction enzymes (Thermo Scientific, Waltham, MA, USA). The RFLP reaction mixture consisted of 1x Buffer Tango, PCR products, deionized water (dH_2_O), and 5 units of the relevant restriction enzyme. The *MMP1* gene amplicon (113 bp) was digested with PdmI (XmnI): the 1G/1G genotype produced fragments of 102 bp and 11 bp, the 1G/2G genotype produced fragments of 113 bp, 102 bp, and 11 bp, and the 2G/2G genotype remained intact (113 bp); the 11 bp fragment was not detected on the gel due to its low molecular weight. The *MMP2* amplicon (391 bp) was digested using HinfI: the mutant TT genotype produced fragments of 338 bp and 53 bp, the CT genotype produced fragments of 391 bp, 338 bp, and 53 bp, and the CC genotype remained intact (the 53 bp fragment was not visible on the gel). The *MMP3* amplicon (129 bp) was digested with PsyI (Tth111I): The 5A/5A genotype yielded a single band at 96 bp, the 5A/6A genotype resulted in two fragments of 129 bp and 96 bp, and the 6A/6A genotype remained undigested at 129 bp. All products were separated by agarose gel electrophoresis under a constant electrical current (20 mA) (Minicell Primo EC 320, Cleaver Scientific for electrophoresis tank and EC-105, Cleaver Scientific MP300 V for power source). The digested bands were stained with fluorescent dyes and analyzed under an ultraviolet transilluminator imaging system. Genotype scores were validated by two independent researchers blinded to the participants’ clinical conditions (Supplemental Files S1, S2, S6–S8).

### 4.5. Statistical Analysis

Continuous variables are expressed as mean ± standard deviation for normally distributed data; and as median and interquartile range (IQR, 25–75% percentiles) for non-normally distributed data. Categorical variables, including genotype and allele distributions, are presented as absolute frequencies and percentages (n, %). Deviations from HWE for the genotype distributions of *MMP1* (−1607 1G/2G), *MMP2* (−735 C/T), and *MMP3* (−1171 5A/6A) were evaluated for both the patient (n = 91) and healthy control (n = 91) groups. Conformity to HWE was assessed by comparing the observed and expected genotype frequencies using the Pearson chi-square test with 1 degree of freedom (df = 1). Statistical analyses were performed using SPSS version 26.0 (IBM Corp., Armonk, NY, USA), and a *p*-value > 0.05 was considered to indicate that the genotype distribution was in HWE. To evaluate the relationship between single nucleotide variations and the risk of developing Peyronie’s disease, the allele and genotype frequencies in the case and control groups were compared using the Pearson chi-square test or Fisher’s exact test when expected cell counts were insufficient. Odds Ratio (OR) and 95% confidence intervals (95% CI) for disease susceptibility were calculated using unconditional binary logistic regression analysis to evaluate codominant, dominant, and recessive genetic patterns. Subgroup analyses were conducted within the patient cohort to examine the effect of genetic variations on specific clinical phenotypes. The association of *MMP* genotypes with categorical clinical parameters (the presence/absence of palpable penile plaque and clinical disease stages) was analyzed using 3 × 2 Pearson chi-square contingency tables. To compare continuous clinical parameters (such as symptom duration) among the three genotype groups, the non-parametric Kruskal–Wallis H test was used due to the skewed distribution of the data; subsequently, a post hoc Dunn test with Bonferroni correction was applied for pairwise comparisons to identify specific genotypic differences. Furthermore, to account for multiple subgroup comparisons and minimize the risk of Type I error inflation, multiplicity adjustments (such as Bonferroni-adjusted p-values where appropriate) were considered for secondary phenotype evaluations. All statistical tests were conducted using two-tailed methods, and *p*-values less than 0.05 (*p* < 0.05) were considered statistically significant.

## 5. Conclusions

The present study demonstrates that *MMP1*, *MMP2*, and *MMP3* gene promoter variations are significantly associated with susceptibility to Peyronie’s disease and provide valuable clinical insight into the disease course, including temporal stage, symptom duration, and plaque presentation. We observed significant differences in the frequencies of specific *MMP* alleles between the patient and control groups. For the *MMP1* (−1607 1G/2G) variation, the frequency of the 2G allele was significantly higher among patients, with logistic regression analyses indicating that the 2G/2G genotype is associated with 3.5-fold higher odds of Peyronie’s disease. Similarly, the *MMP2* (−735 C/T) C allele predominated in the patient cohort, where the CC genotype was associated with approximately three times higher odds of the condition, whereas the TT genotype showed an inverse association with lower odds of disease. For the *MMP3* (−1171 5A/6A) variation, the 5A/5A genotype exhibited the strongest association with disease susceptibility. 

Beyond disease development, these genetic variations demonstrate robust statistical associations with clinical phenotypic characteristics. A highly significant association was identified between the *MMP2* CC genotype and the presence of penile plaques, whereas *MMP1* and *MMP3* genotypes showed no direct association with plaque presence. Furthermore, genotype distributions were strongly correlated with temporal disease presentation. Patients carrying the high-expression-associated *MMP1* 2G/2G, *MMP2* CC, and *MMP3* 5A/5A or 5A/6A genotypes presented characteristically within the active, highly inflammatory acute phase. Conversely, patients in the chronic phase predominantly exhibited genotypes associated with prolonged, indolent courses (*MMP1* 1G/1G, *MMP2* TT, and *MMP3* 6A/6A).

Non-parametric temporal analyses confirmed these patterns, showing significantly compressed median symptom durations in carriers of the *MMP1* 2G/2G, *MMP2* CC, and *MMP3* 5A alleles, while individuals with the *MMP3* 6A/6A genotype experienced significantly extended median durations (*p* < 0.001, adj *p* < 0.001). While these variations align with literature-based hypotheses regarding matrix remodeling dynamics, because our cross-sectional design relied strictly on peripheral blood DNA genotyping without direct tissue-level expression or enzymatic quantification, these genotype–phenotype links represent robust statistical associations rather than direct causal evidence. Altogether, while future large-scale, prospective longitudinal studies and tissue-level expression analyses are essential for validation, these findings establish a strong baseline for understanding genetic susceptibility factors and improving future clinical risk stratification in Peyronie’s disease.

## Figures and Tables

**Table 1:** Allele frequencies for *MMP* gene variations in Peyronie patients and healthy control groups.

Gene Variations	Patient Group (n = 91)			Control Group (n = 91)		
	Allele	Case	Frequency	Allele	Case	Frequency
***MMP1*** **(−1607 1G/2G)**	**1G**	94	0.5170	**1G**	139	0.7640
	**2G**	88	0.4830	**2G**	43	0.2360
	**Total**	182	1.0000	**Total**	182	1.0000
	Hardy–Weinberg Equilibrium Test: Pearson chi2 = 0.525 Pr = 0.4687 ^a^			Hardy–Weinberg Equilibrium Test: Pearson chi2 = 2.878 Pr = 0.0897 ^a^		
***MMP2*** **(−735 C/T)**	**C**	131	0.7200	**C**	89	0.4890
	**T**	51	0.2800	**T**	93	0.5110
	**Total**	182	1.0000	**Total**	182	1.0000
	Hardy–Weinberg Equilibrium Test: Pearson chi2 = 2.201 Pr = 0.1379 ^a^			Hardy–Weinberg Equilibrium Test: Pearson chi2 = 3.163 Pr = 0.0753 ^a^		
***MMP3*** **(−1171 5A/6A)**	**5A**	91	0.5000	**5A**	42	0.2040
	**6A**	91	0.5000	**6A**	140	0.7960
	**Total**	182	1.0000	**Total**	182	1.0000
	Hardy–Weinberg Equilibrium Test: Pearson chi2 = 0.890 Pr = 0.3455 ^a^			Hardy–Weinberg Equilibrium Test: Pearson chi2 = 0.746 Pr = 0.3877 ^a^		

^a^ Hardy–Weinberg Equilibrium test; * significance (*p* < 0.05).

**Table 2:** Logistic regression analysis, odds ratio values of *MMP* gene variations genotype distributions for Peyronie patient and healthy control groups.

Genotype Distributions		Patient Group (n = 91)	Control Group (n = 91)	*p*
***MMP1*** **(−1607 1G/2G)**	1G/1G	26 (28.6%)	56 (61.5%)	<0.001 ^a^*
	1G/2G	42 (46.1%)	27 (29.7%)	
	2G/2G	23 (25.3%)	8 (8.8%)	
***MMP2*** **(−735 C/T)**	CC	50 (54.9%)	26 (28.6%)	<0.001 ^a^*
	CT	31 (34.1%)	37 (40.7%)	
	TT	10 (11.0%)	28 (30.7%)	
***MMP3*** **(−1171 5A/6A)**	5A/5A	25 (27.5%)	8 (8.8%)	<0.001 ^a^*
	5A/6A	41 (45.0%)	26 (28.6%)	
	6A/6A	25 (27.5%)	57 (62.6%)	
**Genotype Distributions**		**Patient Group (n = 91) and Control Group (n = 91)**		***p***
***MMP1*** **(−1607 1G/2G)**	1G/1G	OR: 0.250 (0.134–0.465)		<0.001 ^b^*
	1G/2G	OR: 2.032 (1.104–3.740)		0.023 ^b^*
	2G/2G	OR: 3.509 (1.476–8.343)		0.005 ^b^*
***MMP2*** **(−735 C/T)**	CC	OR: 3.049 (1.649–5.635)		<0.001 ^b^*
	CT	OR: 0.754 (0.413–1.377)		0.358 ^b^
	TT	OR: 0.278 (0.126–0.614)		0.002 ^b^*
***MMP3*** **(−1171 5A/6A)**	5A/5A	OR: 3.930 (1.664–9.280)		0.002 ^b^*
	5A/6A	OR: 2.050 (1.109–3.789)		<0.022 ^b^*
	6A/6A	OR: 0.226 (0.121–0.423)		<0.001 ^b^*

^a^ Chi-square test; ^b^ logistic regression; * significance (*p* < 0.05). **OR**: odds ratio; **1G/1G**: 1Guanine/1Guanine; **1G/2G**: 1Guanine/2Guanine; **2G/2G**: 2Guanine/2Guanine; **5A/5A**: 5Adenine/5Adenine; **5A/6A**: 5Adenine/6Adenine; **6A/6A**: 6Adenine/6Adenine; **CC**: Cytosine-Cytosine; **CT**: Cytosine-Thymine; **TT**: Thymine-Thymine.

**Table 3:** *MMP* gene variations genotype distributions according to plaque presence/absence for Peyronie patients.

Genotype Distributions		Total Patients (n = 91)	Plaque Presence (n = 78)	Plaque Absence (n = 13)	*p*
***MMP1*** **(−1607 1G/2G)**	1G/1G	26 (100.0%)	23 (88.5%)	3 (11.5%)	0.852 ^a^
	1G/2G	42 (100.0%)	35 (83.3%)	7 (16.7%)	
	2G/2G	23 (100.0%)	20 (87.0%)	3 (13.0%)	
***MMP2*** **(−735 C/T)**	CC	50 (100.0%)	45 (90.0%)	5 (10.0%)	<0.001 ^a^*
	CT	31 (100.0%)	29 (93.5%)	2 (6.5%)	
	TT	10 (100.0%)	4 (40.0%)	6 (60.0%)	
***MMP3*** **(−1171 5A/6A)**	5A/5A	25 (100.0%)	23 (92.0%)	2 (8.0%)	0.536 ^a^
	5A/6A	41 (100.0%)	35 (85.4%)	6 (14.6%)	
	6A/6A	25 (100.0%)	20 (80.0%)	5 (20.0%)	

^a^ Chi-square test; * significance (*p* < 0.05, including multiplicity adjustments where applicable); 1G/1G: 1Guanine/1Guanine; 1G/2G: 1Guanine/2Guanine; 2G/2G: 2Guanine/2Guanine; 5A/5A: 5Adenine/5Adenine; 5A/6A: 5Adenine/6Adenine; 6A/6A: 6Adenine/6Adenine; CC: Cytosine-Cytosine; CT: Cytosine-Thymine; TT: Thymine-Thymine.

**Table 4:** *MMP* gene variations genotype distributions according to disease stages for Peyronie patients.

Genotype Distributions		Total Patients (n = 91)	Acute Phase (≤12 months)	Chronic Phase (>12 months)	*p*
***MMP1*** **(−1607 1G/2G)**	1G/1G	26 (100.0%)	19 (73.1%)	7 (26.9%)	0.026 ^a^*
	1G/2G	42 (100.0%)	37 (88.1%)	5 (11.9%)	
	2G/2G	23 (100.0%)	23 (100.0%)	0 (0.0%)	
***MMP2*** **(−735 C/T)**	CC	50 (100.0%)	50 (100.0%)	0 (0.0%)	0.001 ^a^*
	CT	31 (100.0%)	29 (93.5%)	2 (6.5%)	
	TT	10 (100.0%)	6 (60.0%)	4 (40.0%)	
***MMP3*** **(−1171 5A/6A)**	5A/5A	25 (100.0%)	25 (100.0%)	0 (0.0%)	<0.001 ^a^*
	5A/6A	41 (100.0%)	41 (100.0%)	0 (0.0%)	
	6A/6A	25 (100.0%)	16 (64.0%)	9 (36.0%)	

^a^ Fisher’s exact test; * significance (*p* < 0.05, including multiplicity adjustments where applicable); 1G/1G: 1Guanine/1Guanine; 1G/2G: 1Guanine/2Guanine; 2G/2G: 2Guanine/2Guanine; 5A/5A: 5Adenine/5Adenine; 5A/6A: 5Adenine/6Adenine; 6A/6A: 6Adenine/6Adenine; CC: Cytosine-Cytosine; CT: Cytosine-Thymine; TT: Thymine-Thymine.

**Table 5:** *MMP* gene variations genotype distributions according to symptom duration for Peyronie patients.

Genotype Distributions		Total Patients (n = 91)	Mean ± Standard Deviation (Months)	Median (IQR) (Months)	*p*
***MMP1*** **(−1607 1G/2G)**	1G/1G	26 (100.0%)	19.92 ± 23.36	12.00 (5.25–36.00)	0.016 ^a^*
	1G/2G	42 (100.0%)	9.21 ± 9.17	8.00 (4.00–12.00)	
	2G/2G	23 (100.0%)	6.43 ± 3.82	6.00 (3.00–9.00)	
***MMP2*** **(−735 C/T)**	CC	50 (100.0%)	6.46 ± 3.86	6.00 (3.00–12.00)	0.002 ^a^*
	CT	31 (100.0%)	10.13 ± 9.17	8.00 (5.00–12.00)	
	TT	10 (100.0%)	26.50 ± 23.32	24.00 (4.00–48.00)	
***MMP3*** **(−1171 5A/6A)**	5A/5A	25 (100.0%)	6.00 ± 3.81	6.00 (3.50–12.00)	<0.001 ^a^*
	5A/6A	41 (100.0%)	5.68 ± 4.09	4.00 (3.00–9.50)	
	6A/6A	25 (100.0%)	28.52 ± 22.84	24.00 (7.50–54.00)	

^a^ Kruskal–Wallis test; * significance (*p* < 0,05, including post hoc Dunn–Bonferroni multiplicity adjustments); **1G/1G**: 1Guanine/1Guanine; **1G/2G**: 1Guanine/2Guanine; **2G/2G**: 2Guanine/2Guanine; **5A/5A**: 5Adenine/5Adenine; **5A/6A**: 5Adenine/6Adenine; **6A/6A**: 6Adenine/6Adenine; **CC**: Cytosine-Cytosine; **CT**: Cytosine-Thymine; **TT**: Thymine-Thymine.

## Data Availability

The data presented in the present study are available on request from the corresponding author. The data are not publicly available due to ethical reasons.

## Supplementary Materials

The following supporting information can be downloaded at: https://www.mdpi.com/article/10.3390/ijms27167194/s1.

## Abbreviations

The following abbreviations are used in this manuscript:

MMP-1	Matrix Metalloproteinase-1
MMP-2	Matrix Metalloproteinase-2
MMP-3	Matrix Metalloproteinase-3
PCR	Polymerase Chain Reaction
RFLP	Restriction Fragment Length Polymorphism
EDTA	Ethylenediaminetetraacetic Acid
TIMPs	Tissue Metalloproteinase Inhibitors
HWE	Hardy–Weinberg Equilibrium
OD	Optical Density
