# Supplementary material for: Impact of MMP1, MMP2, and MMP3 Gene Variations on Susceptibility to Peyronie’s Disease and Clinical Progression
Source: Int J Mol Sci. 2026 Aug 12;27(16):7194. doi: 10.3390/ijms27167194 (PMC13512950; doi:10.3390/ijms27167194)
Supplement: Supplementary file 1 [file ijms-27-07194-s001.zip › Supplemental File S1 Revise.pdf]

Supplemental File S1. *MMP* gene variations primer series, PCR conditions and restriction enzymes

Gene Variations	*Primer series*	*PCR Conditions*	*RE*
***MMP1* (-1607 1G/2G)**	**FP:** 5′-TCGTGAGAATGTCTTCCCATT-3′<br>**RP:** 5′-TCTTGGATTGATTTGAGATAAGTCAT ATC-3′	5 minutes at 94^{0}C<br>20 seconds at 94^{0}C, 20 seconds at 55^{0}C, 20 seconds at 72^{0}C — 35cycle<br>5 minutes at 72^{0}C	***PdmI (XmnI)***
***MMP2* (-735 C/T)**	**FP:** 5′-GGATTCTTGGCTTGGCGCAGGA-3′<br>**RP:** 5′-GGGGGCTGGGTAAAATGAGGCTG-3′	5 minutes at 94^{0}C<br>45 seconds at 95^{0}C, 45 seconds at 65^{0}C, 45 seconds at 72^{0}C — 35cycle<br>10 minutes at 72^{0}C	***HinfI***
***MMP3* (-1171 5A/6A)**	**FP:** 5′-CTTCCTGGAATTCACATCACTGCCACCACT-3′<br>**RP:** 5'-GGTTCTCCATTCCTTTGATGGGGGGAAAAA-3'	3 minutes at 94^{0}C<br>50 seconds at 94^{0}C, 50 seconds at 58^{0}C, 20 seconds at 72^{0}C — 35cycle<br>5 minutes at 72^{0}C	***PsyI (Tth111I)***

FP: Forward primer; **RP:** Reverse primer; **PCR:** Polymerase chain reaction; **RE:** Restriction enzyme
