# Supplementary material for: Impact of MMP1, MMP2, and MMP3 Gene Variations on Susceptibility to Peyronie’s Disease and Clinical Progression
Source: Int J Mol Sci. 2026 Aug 12;27(16):7194. doi: 10.3390/ijms27167194 (PMC13512950; doi:10.3390/ijms27167194)
Supplement: Supplementary file 1 [file ijms-27-07194-s001.zip › Supplemental File S2 Revise.pdf]

Supplemental File S2: Genotype distributions, PCR-RFLP components, product lengths for *MMP* gene variations

<table>
<tr><th>Gene Variation</th><th>Target Region (rsID)</th><th>PCR Product Lengths</th><th>Genotype Distributions Product Lengths</th></tr>
<tr><td>MMP1
(-1607 1G/2G)</td><td>rs1799750</td><td>113bp</td><td>1G/1G genotype: 102bp, 11bp
1G/2G genotype: 113bp, 102bp, 11bp
2G/2G genotype: 113bp, 11bp
(11bp is not observed).</td></tr>
<tr><td>MMP2
(-735 C/T)</td><td>rs2285053</td><td>391bp</td><td>TT genotype: 338bp, 53bp
CT genotype: 391bp, 338bp, 53bp
CC genotype: 391bp
(53bp is not observed).</td></tr>
<tr><td>MMP3
(-1171 5A/6A)</td><td>rs3025058</td><td>129bp</td><td>5A/5A genotype: 96bp
5A/6A genotype: 129bp, 96bp
6A/6A genotype: 129bp</td></tr>
<tr><td>Gene Variation</td><td colspan="2">PCR Components</td><td>RFLP Components</td></tr>
<tr><td>MMP1
(-1607 1G/2G)</td><td colspan="2" rowspan="3">50 nanograms of isolated DNA
0.2 mM dNTP for each
forward primer and reverse primer
1 x PCR buffer
3mM $MgCl_2$
1.25 Unite Taq DNA polymerase</td><td rowspan="3">1 x Buffer Tango
PCR reaction products
dH_2O
5 Units of restriction enzyme</td></tr>
<tr><td>MMP2
(-735 C/T)</td></tr>
<tr><td>MMP3
(-1171 5A/6A)</td></tr>
</table>

CC: Cytosine-Cytosine; **CT:** Cytosine-Thymine; **TT:** Thymine-Thymine; **1G/1G:** 1Guanine/1Guanine; **1G/2G:** 1Guanine/2Guanine; **2G/2G:** 2Guanine/2Guanine; **5A/5A:** 5Adenine/5Adenine; **5A/6A:** 5Adenine/6Adenine; **6A/6A:** 6Adenine/6Adenine; PCR: Polymerase chain reaction
